# Subglottic Hemangioma: A Hidden Mass Presenting in an Unusual Age Group

**DOI:** 10.7759/cureus.38293

**Published:** 2023-04-29

**Authors:** Thilaga Rajendran, Mawaddah Azman

**Affiliations:** 1 Otolaryngology - Head and Neck Surgery, Faculty of Medicine, Hospital Universiti Kebangsaan Malaysia, Kuala Lumpur, MYS

**Keywords:** adult hemangioma, larynx, subglottis, subglottic hemangioma, hemangioma

## Abstract

Subglottic hemangiomas are rare in adulthood. The presence of the lesion in the subglottic region makes it even more unusual. Moreover, these lesions do not have a typical course and involution changes as seen in the infantile forms. An elderly female initially came with a brief history of dyspnea and symptoms of upper respiratory tract infection. The patient also complained of a change of voice and noisy breathing, with a recent history of intubation following COVID-19 pneumonia and late-onset bronchial asthma. Flexible nasopharyngolaryngoscopy showed a mass below the vocal folds, which was seen to arise from the posterior subglottic region. The patient eventually underwent endoscopic excision of the lesion under general anesthesia and recovered well. Symptoms of hoarseness and stridor, along with a history of intubation, should raise a high index of suspicion for laryngeal diseases. A delay in the diagnosis of an obstructing lesion in the subglottis occurs in the presence of a confounding lung infection and overlap of clinical features with those of bronchial asthma. Surgical excision is required not only to alleviate obstructive symptoms but also to rule out malignancy.

## Introduction

Hemangioma is the most common congenital benign tumor of the larynx, which is rare in adults. It is a vascular malformation that is slow-growing and self-limiting in nature. Hemangiomas can be classified either by the age of occurrence (there are infantile and adult forms), their anatomical location, or their histological characteristics. In their infantile form, these lesions demonstrate a characteristic clinical course marked by rapid proliferation, slow and spontaneous involution, and then resolution [1]. As such, the head and neck region contributes to about 83% of infantile hemangioma cases [2]. The subglottic region is the commonest site for infantile laryngeal hemangiomas. Respiratory distress is more pronounced in children with subglottic hemangiomas, as the subglottis is the narrowest site for a pediatric airway [1]. About 50% of these children tend to present with a co-existing cutaneous hemangioma [3].

Meanwhile, in adults, hemangiomas act rather differently and do not follow the usual phases that occur in children. The lesions are usually non-progressive. In adults, the most frequent location for laryngeal hemangiomas is the supraglottic region (80%), followed by the glottic and subglottic regions [4]. These vascular lesions can occur at any age in adulthood, but men are more likely to be affected. Most of these patients require surgical intervention compared to their counterparts in the pediatric age group [1].

Hereby, we would like to share our experience in dealing with a very rare case of subglottic hemangioma presenting in an elderly female with a co-existing lung infection and bronchial asthma.

## Case presentation

**Figure 1:**
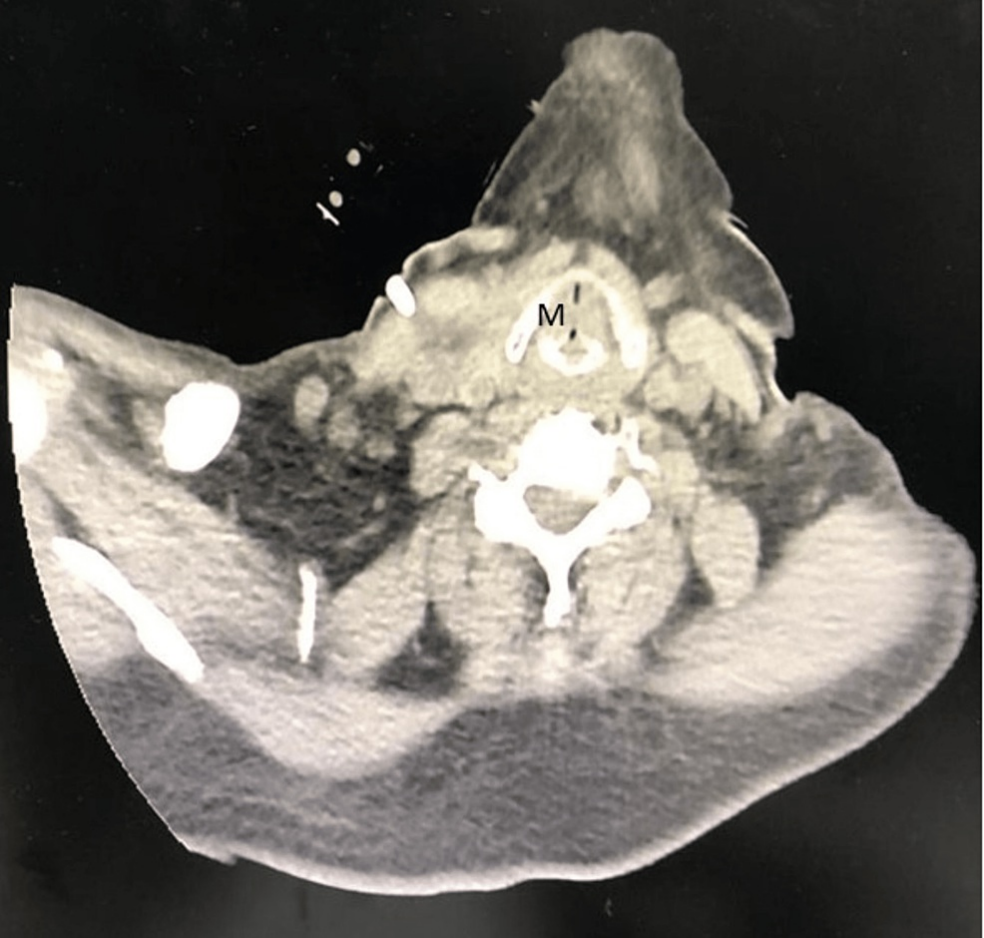
CT of the neck showing a well-defined hypodense submucosal lesion (M) with peripheral enhancement at the posterior subglottic region.

**Figure 2:**
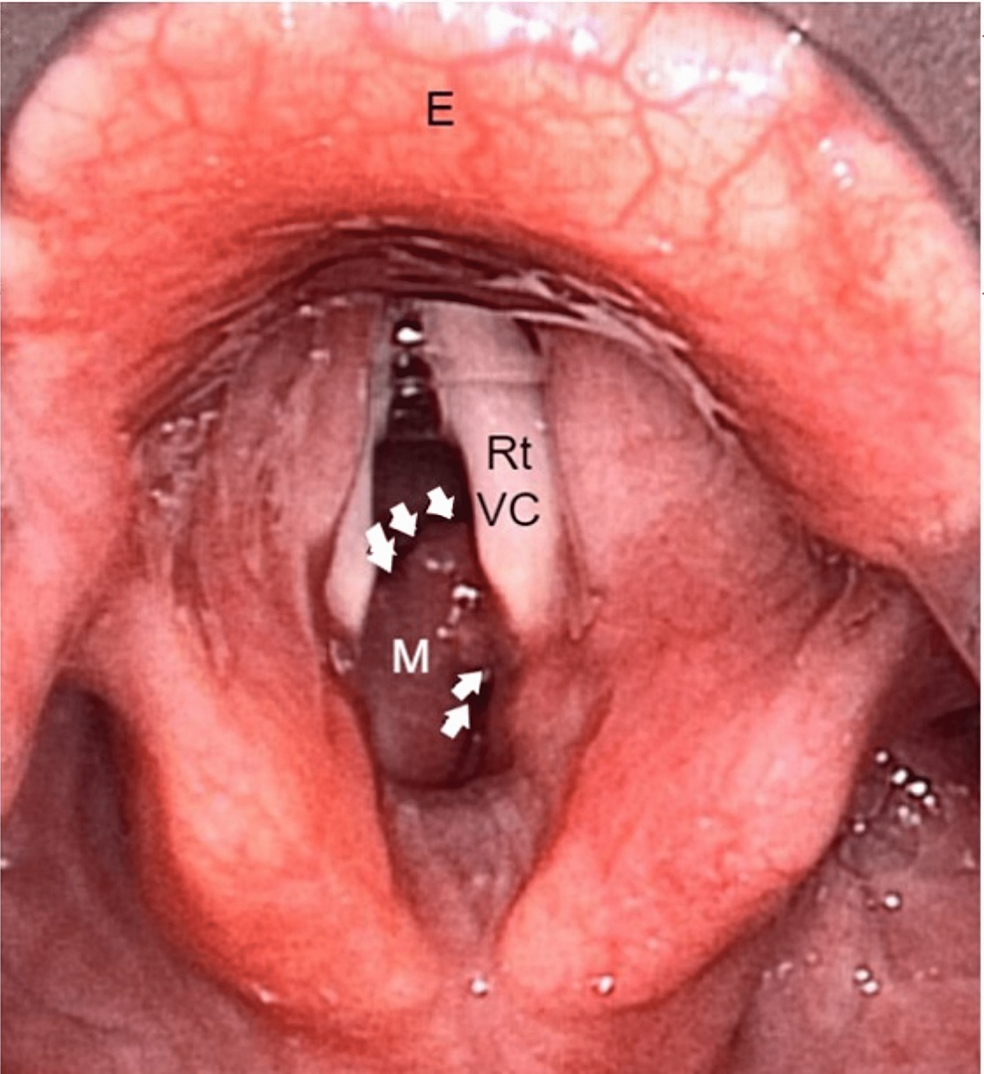
Suspension laryngoscopy showing the pinkish subglottic mass (M) seen beneath the right vocal cord (Rt VC). The white arrows show the outline of the subglottic mass (M) causing significant luminal narrowing of the airway. Epiglottis (E) is also seen.

**Figure 3:**
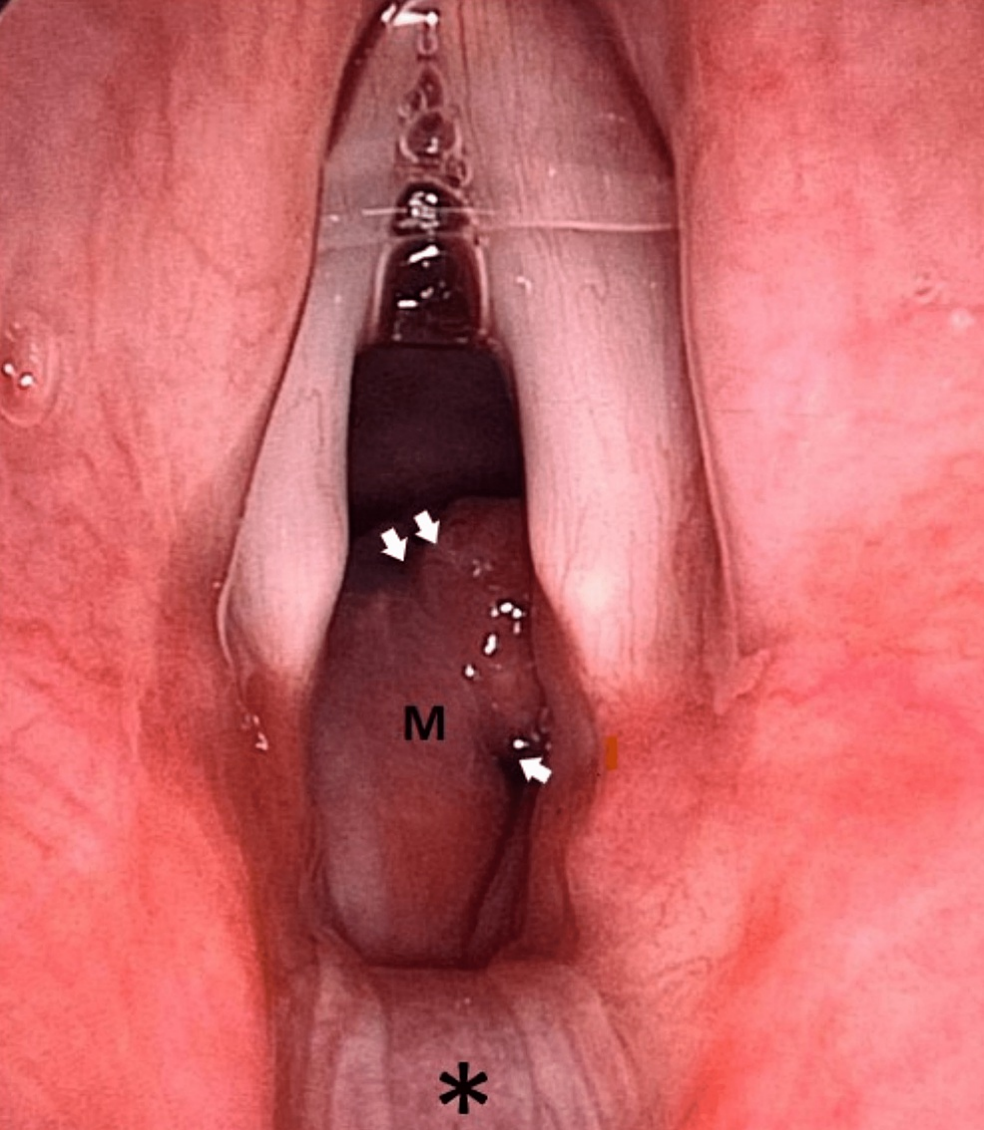
Suspension laryngoscopy showing a close-up view of the subglottic mass (M) arising from the posterior subglottic region (white arrows). Posterior commissure and interarytenoid mucosa appear normal (*).

**Figure 4:**
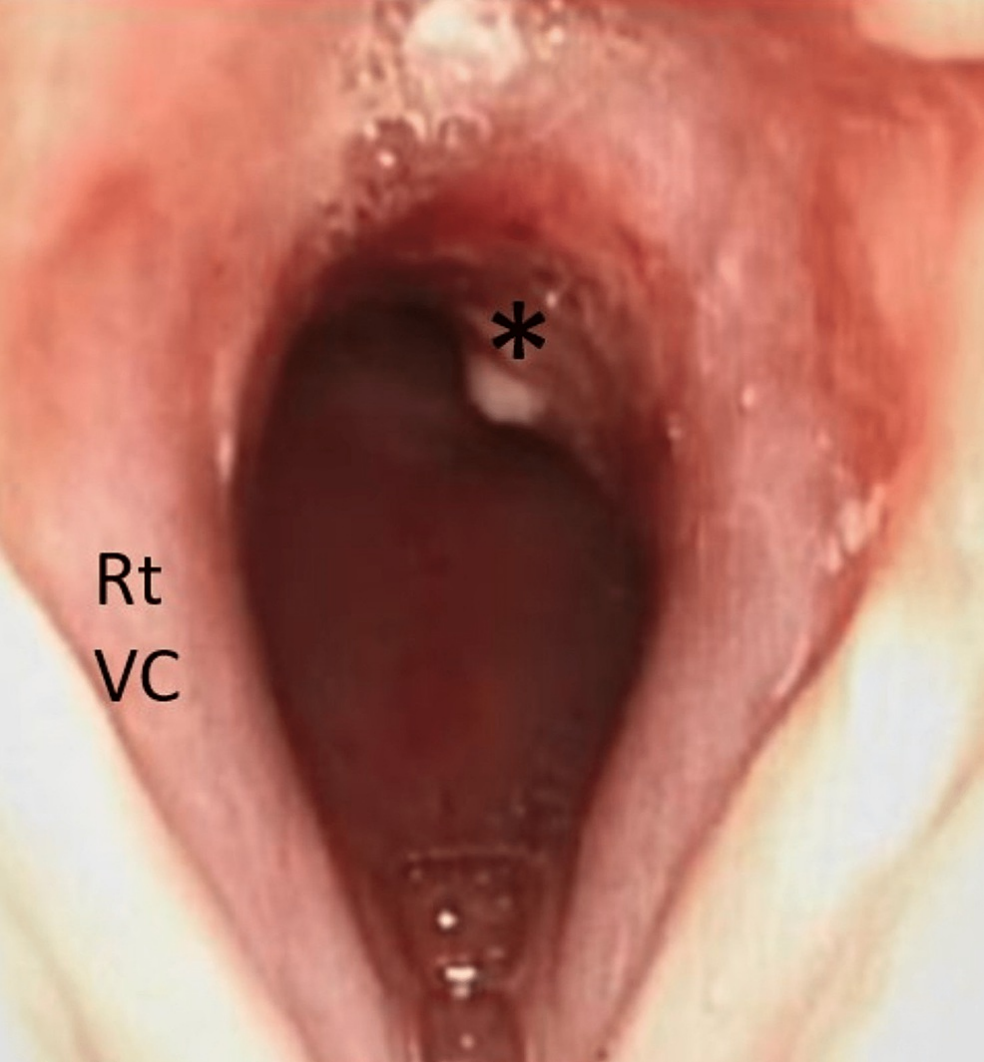
Flexible video laryngoscopic view of the subglottic region (Rt VC: right vocal cord) at postoperative day 14 showing significant improvement of the airway and pale granulation tissue (*) at the left posterolateral wall.

A 73-year-old lady with multiple comorbidities presented to the casualty with a brief history of difficulty breathing for a day. She also had symptoms of an upper respiratory tract infection. The patient also complained of a change of voice for the past month. The voice changes developed post-extubation, where she was electively intubated for five days and kept sedated in the intensive care unit due to coronavirus disease 2019 (COVID-19; CAT-5b) pneumonia. There was no history of change of voice or hemoptysis prior to that. She was admitted to the medical ward and treated for community-acquired pneumonia with concomitant newly diagnosed bronchial asthma. The patient was then referred to the otorhinolaryngology team on the second day of admission for upper airway assessment. Upon assessment, there was soft biphasic stridor with no signs of respiratory distress, and a flexible nasopharyngolaryngoscopy (FNPLS) showed a reddish mass below the vocal folds, which was seen to arise from the posterior subglottic region. The supraglottic structures and vocal folds were normal. In view of a recent history of intubation, the initial diagnosis of acquired subglottic stenosis was made. The patient was started on nebulized dexamethasone and ciprofloxacin 12 hourly, intravenous dexamethasone eight hourly, and intravenous cefepime to treat underlying pneumonia. In view of the non-resolving symptoms, the patient underwent computed tomography (CT) of the neck, which reported a well-defined hypodense submucosal lesion with peripheral enhancement at the left posterior paramidline subglottic region, measuring approximately 0.7 x 1.1 x 1.0 cm. The adjacent cricoid cartilage was eroded with no extralaryngeal extension or suggestion of lymphatic metastasis (Figure 1). The submucosal lesion caused significant luminal narrowing, with the narrowest segment measuring 5.7 mm. The patient was then subjected to tracheostomy under local anesthesia, direct laryngoscopy, and examination under anesthesia. The subglottic mass that was soft, non-vascular, lobulated, and smooth-surfaced was seen arising beneath the right vocal fold (Figures 2, 3). The subglottic mass was excised endoscopically using cold instruments. During the manipulation, pus was aspirated from the mass. The subglottic stenosis significantly improved from Cotton-Myer grade III (75% occlusion of the airway) to grade I (15% occlusion of the airway). Histopathological findings reported the submucosal mass as a lobular capillary hemangioma. Post-operatively, the patient recovered well without any bleeding, and the patient's tracheostomy tube was decannulated on the third postoperative day. A repeat FNPLS showed normal vocal folds with the resolution of the subglottic mass (Figure 4). The patient was seen at the one-month follow-up with no residual or recurrence of symptoms.

## Discussion

Adult subglottic hemangiomas are scarcely reported in the literature. The common attributable factors for laryngeal hemangiomas are cigarette smoking, vocal abuse, and laryngeal trauma such as intubation [5]. In this case report, the patient had a brief history of intubation about one month prior to her symptom of change of voice. Upper airway obstruction is a life-threatening emergency that needs urgent intervention. Though the initial diagnoses of this patient discussed are pneumonia and newly diagnosed bronchial asthma, other causes of upper airway obstruction need to be kept in mind in dealing with such cases. Any unresolved symptoms, such as respiratory distress or stridor, should be immediately investigated to prevent fatal complications. Symptoms such as hoarseness and stridor may indicate the location of the lesion, which could be present at any of the laryngeal subsites at either supraglottic, glottic, or subglottic levels. Thus, FNPLS investigation is paramount in detecting the location of the laryngeal hemangioma, as the subglottic region can be quite difficult to visualize. The etiology of upper airway obstruction can be classified as mechanical, traumatic, infectious, inflammatory, or iatrogenic. The case discussed here has a mechanical cause [6].

Another important differential diagnosis that ought to be considered in any adult with stridor or upper airway obstruction is laryngeal carcinoma, especially in the elderly group. Thus, surgical intervention is necessary not only to alleviate the symptoms suffered but also to attain a histopathological diagnosis to distinguish the lesion from any malignancy. In laryngeal carcinoma, the main symptom is also hoarseness, as experienced by the patient discussed in this case report. Other significant symptoms include a persistent sore throat, dysphagia, and dyspnea. The duration of each of the symptoms can also differ. According to a retrospective study done in Finland in 1999, hoarseness generally has the longest duration of any symptom, whereas more worrying symptoms like dyspnea and hemoptysis have the shortest durations [7].

The management of laryngeal hemangiomas in adults is mostly surgical, as these vascular lesions do not regress spontaneously. Generally, smaller hemangiomas can be treated with laser ablation, corticosteroid injections, and cryosurgery. On the other hand, large lesions with extension to deeper structures require endoscopic laryngeal surgical excision or radiotherapy [6,8]. In this case report, the patient underwent tracheostomy prior to the excision of the lesion. However, the tracheostomy was a temporary measure, given the concomitant existence of lung infection and bronchial asthma.

## Conclusions

Adult subglottic hemangioma is a very rare entity that can occur in patients with hoarseness and respiratory distress. Unresolved co-existing lung infections with a history of intubation should raise a high index of suspicion for these types of rare lesions. Surgical excision is required not only to alleviate obstructive symptoms but also to rule out malignant diseases in the larynx.
